# The Daily and Two-Day Usage of Low-Dose Atropine on Myopic Control in a Low-Myopia Population

**DOI:** 10.3390/jcm14103458

**Published:** 2025-05-15

**Authors:** Chia-Yi Lee, Shun-Fa Yang, Jing-Yang Huang, Ie-Bin Lian, Chao-Kai Chang

**Affiliations:** 1 Institute of Medicine, Chung Shan Medical University, Taichung 40201, Taiwan; 2 Nobel Eye Institute, Taipei 10041, Taiwan; 3 Department of Ophthalmology, Jen-Ai Hospital Dali Branch, Taichung 41265, Taiwan; 4 Department of Medical Research, Chung Shan Medical University Hospital, Taichung 40201, Taiwan; 5 Institute of Statistical and Information Science, National Changhua University of Education, Changhua 50007, Taiwan; 6 Department of Optometry, Yuanpei University of Medical Technology, Hsinchu 30015, Taiwan

**Keywords:** atropine, spherical equivalent refraction, axial length, age, frequency

## Abstract

**Objectives**: The aim of this study was to evaluate the effectiveness of using low-dose atropine (ATR) at different instillation frequencies on myopia control in a low-myopia population. **Methods**: A retrospective cohort study was conducted, and patients using 0.01% ATR and exhibiting a myopia degree ranging from +0.00 to −1.00 diopter (D) were included. A total of 32 and 26 eyes from 32 and 26 individuals were included in the daily group and two-day group, respectively. The main outcomes of this study are the progression of the spherical equivalent refraction (SER) and the elongation of the axial length (AXL). The Mann–Whitney U test and generalized linear model were used to perform the statistical analysis. **Results**: After a follow-up period of one year, the change in SER was similar between the daily group and two-day group (−0.24 ± 0.09 versus −0.26 ± 0.08, *p* = 0.393). In addition, there was an insignificant difference in AXL elongation between the daily group and two-day group (0.09 ± 0.07 versus 0.10 ± 0.09, *p* = 0.655). The trends observed in SER progression (*p* = 0.604) and AXL elongation (*p* = 0.779) were statistically identical between the daily group and the two-day group. **Conclusions**: The results of the two-day use of low-dose ATR regarding SER and AXL control are similar to those with the daily use of low-dose ATR in children with low myopia.

## 1. Introduction

Myopia is a prevalent disease that is experienced by more than 15 percent of people [1]. Partially due to the usage of visual display devices in recent decades, the prevalence of myopia has gradually increased, which is positively correlated with the time of visual display device usage [2,3]. Axial length (AXL) elongation is the main pathophysiology associated with the progression of myopia and can thus be used to accurately monitor it [4]. Other than the reduced visual acuity caused by the negative effects of myopia [5], high myopia can contribute to myopic maculopathy, optic nerve degeneration, glaucoma, and retinal detachment, which should be prevented [6].

Regarding the current treatments able to achieve myopic control, atropine (ATR) has been used for more than 30 years, exhibiting fair outcomes and patient tolerance [7]. Regarding the pharmacokinetics of ATR, ATR is ionized on the ocular surface and can bind to anterior segment tissue and posterior ocular tissues in 5 and 24 h, respectively [8]. The pharmacological effect of ATR begins after 2 h from the administration and can persist up to 14 days [9]. Regarding lens-based myopic control, the orthokeratology contact lenses have shown good spherical equivalent refraction (SER) and AXL control in different populations [10]. More recently, dual-focus contact lenses and defocus incorporated multiple segments (DIMS) spectacle lenses have also been introduced into clinical practice; the effectiveness of these tools for myopic control is not inferior to high-concentration ATR and orthokeratology contact lenses [1,11,12,13]. In addition to monotherapy, the combined usage of ATR and orthokeratology contact lenses revealed a superior myopic control effect compared to the orthokeratology contact lens usage [14].

In clinical practice, ATR is applied once per day, generally before sleep [15]. However, the side effects of ATR include photophobia, grittiness, allergic reactions, and blurry vision at near distances and may reduce the compliance of patients [16]. In patients with moderate myopia, the alternating monocular use of 0.125% ATR has been studied, and it exhibited a fair outcome [17]. Regarding the use of low-dose ATR, the application of 0.01% ATR could effectively reduce SER and AXL progression in the European population, according to a previous study [18]. However, another study proposed that the use of 0.01% ATR could not slow the progression of myopia and AXL elongation compared to the placebo group [19], and that the cessation of 0.01% ATR may cause the rapid progression of myopia [20]. When comparing the use of 0.01% ATR to other low-dose ATR concentrations, it was found that both the application of 0.01% ATR and 0.02% ATR contributed to lower AXL elongations compared to the control group [21]. However, other large-scale studies presented conflicting results concerning the effect of myopic control when using 0.01% ATR compared to ATR at higher concentrations [22,23]. Consequently, the optimum dose and frequency of ATR administration have not been determined. Because the low-myopia population has a lower risk of myopic progression than the high-myopia population [24], modifying the frequency at which ATR is administered in patients with low myopia may be feasible, but this requires further elucidation.

Consequently, the purpose of the current study is to compare the effect of administering low-dose ATR at different frequencies on myopic control in a population with low myopia. The degree of SER progression and AXL elongation was included in the analysis.

## 2. Materials and Methods

### 2.1. Patient Selection

This retrospective cohort study was conducted at the Nobel Eye Institute. The patients were enrolled in the current study (1) if they had a myopia degree ranging from +0.00 diopter (D) to −1.00 D via cycloplegia refraction, (2) if they had been administered 0.01% atropine, (3) if they were less than 10 years of age, and (4) if they did not use other concentrations of ATR and other myopic control tools. Then, the patients were divided according to their frequency of 0.01% ATR administration, namely once every day or once every two days. At 21:00 every day in the daily group, 0.01% ATR was administered, and 0.01% ATR was administered at 21:00 every two days in the two-day group. Only the data from the left eye of each patient were applied in the current study. Finally, a total of 32 and 26 eyes from 32 and 26 individuals were enrolled in the daily group and two-day group, respectively.

### 2.2. Ophthalmic Examination

The initial data of each child, including their age, sex, corrected distance visual acuity (CDVA), sphere degree, cylinder degree, corneal astigmatism, and AXL, were obtained from medical documents. Their manifest refraction and AXL were measured using an autorefractor (KR-8900, Topcon, Itabashi-ku, Tokyo, Japan) and biometry device (IOL Master 500, Carl Zeiss, Göschwitzer Str., Jena, Germany) sequentially. The patients’ initial simulated keratometry and corneal astigmatism were measured using a topographic device (TMS-5, Tomey Corporation, Nishi-Ku, Nagoya, Japan). All the above examinations were performed three times, and the average values of these results were used. The SER was defined as the sphere power plus half of the cylinder power in the current study. In addition, the cycloplegia SER was measured before and after the ATR therapy. Regarding the cycloplegia SER procedures, topical tropicamide (Better eye drop, Aseptic Innovative Medicine Co., Ltd., Taoyuan dist., Taoyuan, Taiwan) was utilized, and then the optometrists examined the pupil diameter and the cycloplegia SER was checked to see whether the pupil diameter was larger than 8 mm. All children/their eyes were measured using the same device and by the same physician during the follow-up period. We used tropicamide because it is the only short-term cycloplegic agent in our institution. The SER and AXL were measured before ATR therapy, 3 months after ATR therapy, 6 months after ATR therapy, 9 months after ATR therapy, and 1 year after ATR therapy. The patients’ compliance with eye drop administration was checked by the physician, and this information was obtained from the children’s parents every one or two months, with all the children obeying the treatment program according to the parents.

### 2.3. Statistical Analysis

The SPSS 20.0 version (SPSS Inc., Chicago, IL, USA) was used for all statistical analyses performed in the current study. The Shapiro–Wilk test was performed, and the non-normality of all the data for the study groups was confirmed (all *p* < 0.05). In addition, the statistical power of the current study was 0.75, with a 0.05 alpha value and a medium effect size, which was generated using G∗Power version 3.1.9.2 (Heinrich Heine Universität at Düsseldorf, Germany). Descriptive analysis was used to present the basic data between the two groups. The Mann–Whitney U test and the Chi-square test were used to compare the baseline characteristics between the two groups. In addition, the Mann–Whitney U test was also used to compare the changes in cycloplegia SER and AXL between the two groups before and one year after ATR treatment. The changes in SER and AXL between the two groups were analyzed using the generalized linear model after adjusting for the initial age, sex, and cycloplegia SER and AXL (the one-day group was used as the reference group). The statistical significance was determined as *p* < 0.05 in the current study, and the *p* value was less than 0.001, denoted as *p* < 0.001.

## 3. Results

The baseline features of the two groups are shown in Table 1. The mean ages were 7.72 ± 1.22 and 7.50 ± 1.32 in the daily group and two-day group, respectively, without a significant difference (*p* = 0.519). In addition, the sex distribution did not reveal a significant difference between the two groups (*p* = 0.562). Concerning the ophthalmic parameters, visual acuity, refraction, corneal parameters, and AXL demonstrated similar values between the daily and two-day groups (all *p* > 0.05) (Table 1).

**Figure 1:**
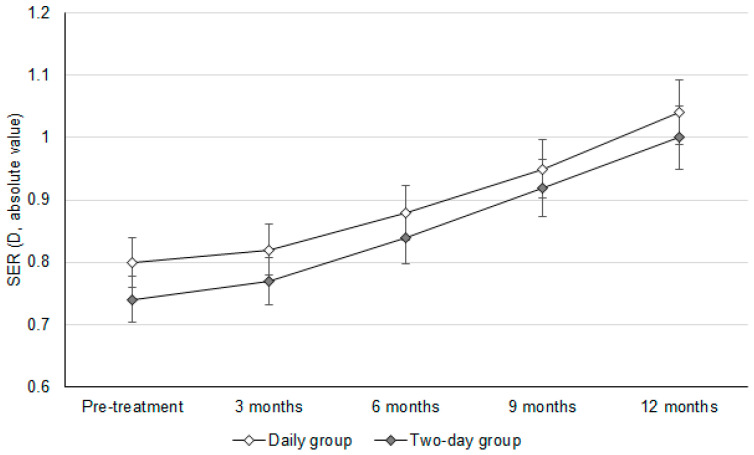
The changes in the spherical equivalent refraction between groups. D: diopter, SER: spherical equivalent refraction.

**Figure 2:**
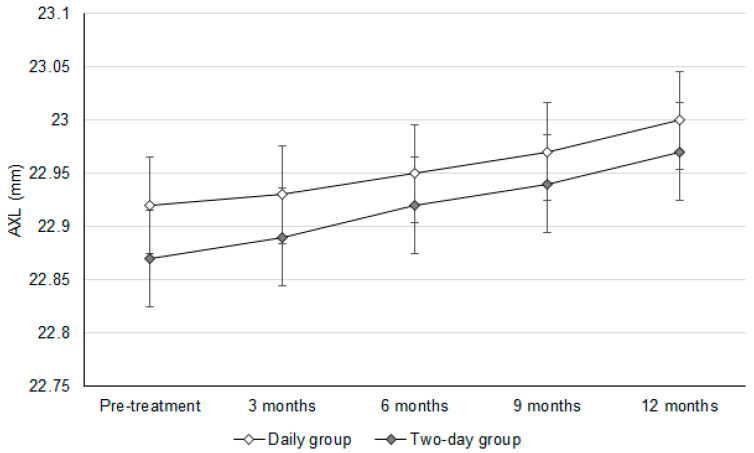
The changes in the axial length between groups. AXL: axial length.

The initial SER values were −0.80 ± 0.11D and −0.74 ± 0.14D in the daily group and two-day group, respectively (*p* = 0.082). In addition, the baseline AXL values were 22.92 ± 0.29 mm and 22.87 ± 0.24 mm in the daily group and two-day group, respectively (*p* = 0.586). After a follow-up period of one year, the change in SER was similar between the daily group and two-day group (−0.24 ± 0.09 versus −0.26 ± 0.08, *p* = 0.393) (Table 2). In addition, there was an insignificant difference in the AXL elongation between the daily group and two-day group (0.09 ± 0.07 versus 0.10 ± 0.09, *p* = 0.655) (Table 2). In addition, the trends observed in SER progression (adjusted odds ratio: 1.010; 95% confidence interval: 0.986–1.033; *p* = 0.604) and AXL elongation (adjusted odds ratio: 1.007; 95% confidence interval: 0.989–1.025; *p* = 0.779) were statistically identical between the daily group and two-day group (Figure 1 and Figure 2).

## 4. Discussion

In the current study, the changes in SER one year after ATR myopia control were similar between the daily group and the two-day group. Moreover, the elongation of AXL also presented similar values between the daily group and the two-day group.

The changes in SER and AXL showed insignificant differences between the daily group and the two-day group in the current study. A previous study showed that low-dose ATR had a fair effect on SER control compared to that without ATR application [25]. In addition, AXL elongation can also be controlled well via the application of low-dose ATR [22]. However, the optimum frequency of low-dose ATR administration for different degrees of myopia has not been fully established. Our findings may provide preliminary evidence that the effects of the daily application of low-dose ATR and the two-day application of low-dose ATR on myopic control are similar in a population with low myopia. Furthermore, the baseline characteristics were statistically identical between the two groups; thus, the influence of baseline characteristics may be minimal. In addition, we adjusted the initial age, sex, and cycloplegia SER and AXL to analyze the trends in SER progression and AXL elongation between groups. However, the trends observed in SER progression and AXL elongation did not differ significantly between the two groups. Consequently, the effectiveness of myopic control via daily low-dose ATR administration and two-day low-dose ATR administration may indeed be comparable. While the main mechanism implicated in the effects of ATR on myopic control is still unclear, the regulation of globe growth via the stimulation of the scleral anticholinergic receptor might represent a possible etiology [26]. In addition, according to a previous study, the half-life of ATR is longer than 24 h [27]. Although the rate of ATR metabolism may differ among individuals, we speculate that the ATR can be retained in the tissue of most Taiwanese people for more than one day. Therefore, the two-day utilization of ATR could contribute to similar myopic control compared to the daily utilization of ATR in a population with low myopia, which is reflected in the changes in SER and AXL observed in the current study. For the potential clinical implications, the long-term usage of low-concentration ATR may induce some complications like reading difficulties, photophobia, mydriasis, and headaches [28]. The complications of ATR, like photophobia, would reduce patient adherence, which is a crucial factor for myopic control effect [29], and overcoming ATR-related side effects is included in the adherence strategy of ATR usage [30]. Thus, the reduction in ATR administration frequency may decrease the rate of complications and elevate patient adherence. On the other hand, the total amount of ATR eye drops would be reduced in two-day usage, and the cost-effectiveness of ATR on myopia may be enhanced. Accordingly, the two-day ATR usage may indeed have benefits in clinical implications.

Regarding the possible confounding factors, other than the baseline characteristics of the study population, hereditary myopia can contribute to the occurrence of myopia and be associated with several congenital defects [31,32]. In this study population, the patients’ family history of myopia was surveyed and obtained from the parents of all the children. According to their medical records, no family history of high myopia was found, and thus, the influence of hereditary myopia might be, if not totally, excluded from the current study. In addition, no congenital disease was found in our study population, except in one child who was diagnosed with glucose-6-phosphate dehydrogenase deficiency. On the other hand, the performance of work requiring close visual proximity to an object, such as the use of visual display devices and reading, is correlated with the development of myopia [15,33]. All the children in the current study reside in the urban area of Taiwan, where visual display devices are always available to children; therefore, parents need to control the time that their children spend using these visual display devices. Moreover, most children (more than 80%) in Taiwan need to go to a cram school after elementary school, and they will keep reading and studying after cram school due to the high emphasis placed on schoolwork in Taiwan. Consequently, the confounding factors associated with myopia may be similar among all the participants in the current study and may not significantly influence the results.

Regarding the myopic control effect observed in the current study compared to previous research, the mean SER progression in the current study was approximately −0.25D after one year of 0.01% ATR treatment. In earlier research, the mean SER progression after the one-year application of 0.01% ATR was −0.26D [25]. In another study analyzing the effects of 0.125% ATR every other night, the mean annual SER progression was −0.29D [34]. Other studies found that the one-year application of 0.01% ATR resulted in SER progression ranging from −0.24D to −0.40D [18,19,20,21]. The myopic control effect observed in the current study may not be inferior to that observed in previous research. On the other hand, in a previous study, the mean AXL elongation was 0.09 mm one year after ATR treatment, and the AXL elongation was approximately 0.20 mm after one year of 0.05% ATR treatment [23]. In addition, another study using 0.01% ATR demonstrated an AXL elongation of 0.75 mm after five years of myopic control [22]. Other studies that administered 0.01% ATR for myopic control demonstrated an AXL elongation ranging between 0.20 mm and 0.25 mm after one year of treatment [18,19,20,21]. Thus, the AXL control effect in the current study is comparable to that observed in previous research on ATR usage. If we compare our results to a previous study that used other myopic control tools, our results show that the SER progression observed was similar to that observed in a previous study analyzing the application of orthokeratology contact lenses [14]. Moreover, the AXL elongation observed in the current study is numerically similar to that observed in a previous article using dual-focus contact lenses [35].

Regarding the myopic control effect of low-concentration ATR in the current study, the AXL elongation after one year of 0.01% ATR treatment was 0.09 and 0.10 mm in the daily group and the two-day group, respectively. The normal eyes showed an AXL elongation rate of about 0.10 to 0.23 mm per year in an age range similar to our study population, according to a previous study [36], and the similar AXL elongations in our study population, which is actually near the lower limit of this range, indicate that the AXL elongations of our patients are similar to the normal eye but not the myopic eye. In further comparison to the previous studies, the annual AXL elongations in their control group, which did not receive low-concentration ATR treatment, ranged from 0.13 to 0.27 mm [18,19,20,21], and the AXL elongations in our patients are numerically lower than in the previous experiences. Accordingly, the myopia control effect of low-dose atropine may exist in the current study, and the myopic control effect might be fair, which may be due to our study population possessing a relatively low myopia degree.

Regarding epidemiology, the prevalence of myopia is increasing worldwide [37]. Among white people, the prevalence of myopia in the United States is approximately 20–40 percent [38]. In the Asian population, the rate of myopia is much higher, with the prevalence of myopia in Hong Kong and Taiwan reaching 80 percent [38]. The management of complications associated with high myopia, including the intravitreal injection of anti-vascular endothelial growth factor and trans pars plana vitrectomy, can incur significant costs [6,15]. To prevent the formation of high myopia, early intervention to reduce myopic progression cannot be over-emphasized, and the usage of low-dose ATR is an important intervention in this field [39]. Accordingly, the effects of low-dose ATR when using different protocols could be reported.

Our study has some limitations. Firstly, the single-center retrospective nature of the current study may reduce the homogeneity of the study population compared to prospective research, although the baseline characteristics between the two groups were similar. In addition, we only measured the presence of keratometry and corneal astigmatism as a baseline evaluation before the administration of 0.01% ATR; after the low-dose ATR treatment, these data were absent. In addition, the number of cases in the current study was small, with only 58 eyes being included and analyzed in the current study. The low case numbers/small sample sizes are reflected in the relatively low statistical power and may contribute to some statistical bias. The absence of cyclopentolate drops at our institution may have reduced the accuracy of cycloplegia refraction in the current study. The potential recall bias may occur in compliance reporting since the compliance was reported by parents rather than being monitored full-time by research staff. Finally, all the children included in the current study were Han Taiwanese; thus, the external validity of the current study may be reduced.

## 5. Conclusions

In conclusion, the daily use of low-dose ATR and two-day use of low-dose ATR result in a similar effect on SER and AXL control in a low-myopia population. Consequently, both the daily and two-day administration of ATR may be considered in children with low myopia. Further large-scale, double-blinded, prospective randomized controlled trials with a longer follow-up period are required to confirm the findings of the current study and evaluate the optimal dosage of low-concentration ATR on myopia control and prevention.

## Figures and Tables

**Table 1:** Pre-treatment data among the study groups.

Characters	Daily Group (N = 32)	Two-Day Group (N = 26)	*p*
Age	7.72 ± 1.22	7.50 ± 1.32	0.519
Sex (male/female)	18:14	15:11	0.562
BCVA (LogMAR)	0.00 ± 0.01	0.00 ± 0.01	0.999
Sphere power	−0.67 ± 0.13	−0.60 ± 0.15	0.082
Cylinder power	−0.25 ± 0.08	−0.28 ± 0.10	0.249
SER	−0.80 ± 0.11	−0.74 ± 0.14	0.082
Simulated keratometry	43.24 ± 1.78	43.52 ± 1.91	0.656
Corneal astigmatism	0.44 ± 0.16	0.52 ± 0.17	0.087
AXL	22.92 ± 0.29	22.87 ± 0.24	0.525

ATR: atropine, AXL: axial elongation, N: number, SER: spherical equivalent refraction.

**Table 2:** Change in spherical equivalent refraction and axial length among the three groups.

Characters	Daily Group	Two-Day Group	*p*
SER			
Pre-treatment	−0.80 ± 0.11	−0.74 ± 0.14	0.082
Post-treatment	−1.04 ± 0.12	−1.00 ± 0.14	0.307
Change	−0.24 ± 0.09	−0.26 ± 0.08	0.393
AXL			
Pre-treatment	22.92 ± 0.29	22.87 ± 0.24	0.525
Post-treatment	23.00 ± 0.23	22.97 ± 0.21	0.689
Change	0.09 ± 0.07	0.10 ± 0.09	0.655

AXL: axial elongation, SER: spherical equivalent refraction.

## Data Availability

The data used in the current study are available from the corresponding author upon reasonable request.

## Abbreviations

The following abbreviations are used in this manuscript:

ATR	atropine
AXL	axial length
CI	confidence interval
D	diopter
N	number
OR	odds ratio
SER	spherical equivalent refraction
